# Development of a human iPSC-derived placental barrier-on-chip model

**DOI:** 10.1016/j.isci.2023.107240

**Published:** 2023-07-13

**Authors:** Agathe Lermant, Gwenaëlle Rabussier, Henriëtte L. Lanz, Lindsay Davidson, Iain M. Porter, Colin E. Murdoch

**Affiliations:** 1 Systems Medicine, School of Medicine, University of Dundee, Dundee DD1 9SY, UK; 2 MIMETAS, Oegstgeest 2342, the Netherlands; 3 Human Pluripotent Stem Cell Facility, School of Life Sciences, University of Dundee, Dundee DD1 5EH, UK; 4 Dundee Imaging Facility, School of Life Sciences, University of Dundee, DD1 5EH, UK

**Keywords:** Pathophysiology, Biotechnology, Cell biology

## Abstract

Although recently developed placenta-on-chip systems opened promising perspectives for placental barrier modeling, they still lack physiologically relevant trophoblasts and are poorly amenable to high-throughput studies. We aimed to implement human-induced pluripotent stem cells (hiPSC)-derived trophoblasts into a multi-well microfluidic device to develop a physiologically relevant and scalable placental barrier model. When cultured in a perfused micro-channel against a collagen-based matrix, hiPSC-derived trophoblasts self-arranged into a 3D structure showing invasive behavior, fusogenic and endocrine activities, structural integrity, and expressing placental transporters. RNA-seq analysis revealed that the microfluidic 3D environment boosted expression of genes related to early placental structural development, mainly involved in mechanosensing and cell surface receptor signaling. These results demonstrated the feasibility of generating a differentiated primitive syncytium from hiPSC in a microfluidic platform. Besides expanding hiPSC-derived trophoblast scope of applications, this study constitutes an important resource to improve placental barrier models and boost research and therapeutics evaluation in pregnancy.

## Highlights

• A 3D perfused placental barrier with high integrity differentiated from hiPSC
• On-chip differentiation improved the barrier spatial organization and directionality
• Detection of endocrine, fusogenic markers and placental transporters
• 3D microfluidic format boosted mechanosensing and cell surface receptor signaling

## Graphical abstract

**Figure:**
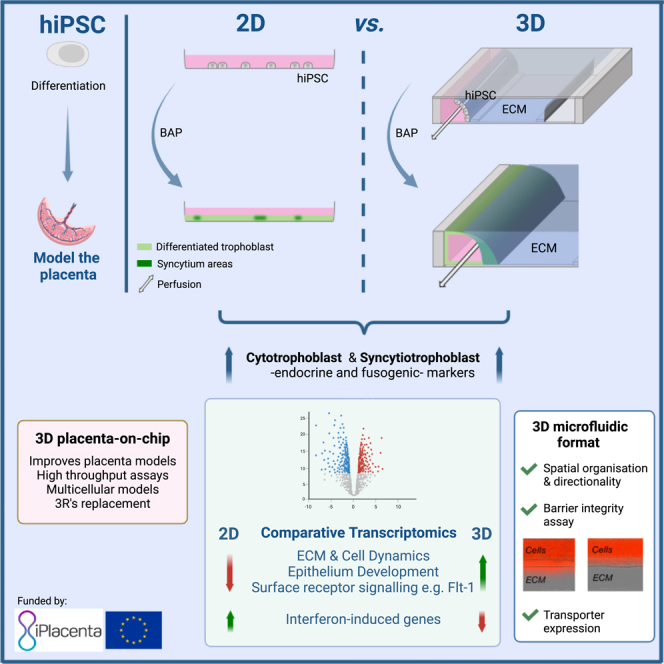

For a Figure360 author presentation of this figure, see https://doi.org/10.1016/j.isci.2023.107240.

## Introduction

The placenta is composed of multi-layer specialized cells from fetal origin working together to support the developing fetus. Shortly after implantation, a layer of actively proliferating cytotrophoblasts (CTB) can either differentiate into syncytiotrophoblasts (STBs) or extravillous trophoblasts (EVTs). STB form a multinucleated outer layer, called syncytium, directly bathing in maternal blood and constituting the area of exchanges between the maternal and fetal blood circulations.^1^ In particular, the syncytium provides an essential and selective barrier regulating fetomaternal transfer of physiologic compounds and xenobiotics, protecting the growing fetus from pathogen entry and secreting hormones necessary for successful pregnancy outcome. EVT are highly migratory trophoblasts involved in the remodeling of maternal spiral arteries.^1^

Developing more relevant human *in vitro* models of the placental barrier is an essential step to improve our ability to conduct placental research as well as provide new methods to assess potential drug toxicity. Several severe pregnancy complications including preeclampsia and intra-uterine growth restriction are associated with dysfunction in the formation of the syncytium or the release of syncytial products such as anti-angiogenic factors.^2^^,^^3^ Likewise, the potential for various drugs, viruses, vaccines, environment pollutants and nanoparticles to cross the placental barrier raised growing concerns and the development of systematic approaches to reliably define the ability of a particular compound to cross the placental barrier has become a topic of interest in the scientific community and pharmaceutical industry.^4^ The *ex vivo* placental perfusion model, although providing a valuable tool for placental research, has a short viability time as artificial perfusion can only be carried out within 2–6 h.^4^ Recent efforts to bridge the gap between 2D cultures and *ex vivo* systems included the use of transwell assays or organoid culture for modeling the placental barrier.^5^^,^^6^^,^^7^^,^^8^ Yet, implementing a dynamic flow remains challenging and these systems are poorly amenable to standardization and high-throughput studies.

The lack of a physiologically relevant, practical, and scalable placental barrier model not only has the potential to hold back research on pregnancy complications but is putting pregnant women and unborn fetuses at risk. The randomized clinical trial STRIDER investigating the clinically safe Sildenafil in pregnancy was halted in 2021 because of fetal deaths, bringing into question the effectiveness of pre-clinical safety assessment for randomized clinical trials in pregnancy and evidencing the pressing need for relevant human models for drug safety and efficacy data in pregnancy.^9^^,^^10^^,^^11^

Microfluidic placenta-on-chip systems are recently emerging as promising alternatives to current placental barrier models because of their microscale format and the possibility to culture cells in a 3D dynamic environment integrating *in vivo*-like biomechanical cues. Especially, the culture of trophoblastic cells in microfluidic conditions promotes the formation of a polarized epithelium at the interface between two accessible compartments, a format particularly well-suited to model the maternal-fetal axis.^12^^,^^13^^,^^14^ The typical design of microfluidic-based placental barrier models consists in two perfused microchannels engineered on a polydimethylsiloxane (PDMS) chip, representing maternal and fetal compartments, separated by a porous membrane.^12^^,^^13^^,^^14^^,^^15^^,^^16^^,^^17^^,^^18^^,^^19^^,^^20^ Other groups have developed more complex versions of placenta-on-chip systems by replicating villi-like geometries, adding microsensor arrays or additional tissue and extracellular matrix (ECM) components in a multilayer format.^21^^,^^22^^,^^23^ Altogether, placenta-on-chip devices have been employed in several studies to examine the transport of glucose, drugs, bacteria, caffeine or environmental nanoparticles across the placental barrier.

Despite the promising perspectives for placental modeling, the field of placenta-on-chip is still in its infancy and the systems developed so far have several avenues for improvement. First, many current placenta-on-chip models lack a physiologically relevant trophoblast component. Most microfluidic placental barrier models use the choriocarcinoma cell lines BeWo or JEG-3 because they have been the only option that can be handled and propagated easily compared to primary trophoblasts.^12^^,^^13^^,^^14^^,^^15^^,^^16^^,^^17^^,^^18^^,^^19^^,^^21^^,^^23^ Yet, their divergence from primary trophoblasts and the inability to associate cancer cell lines with a specific stage of pregnancy complicates the extrapolation of results to human pregnancy.^24^ Moreover, placenta-on-chip models are commonly made on individual chips which either lack perfusion or require perfusion by syringe pumps, a system that is poorly amenable to high-throughput studies.

Perfusable, multi-chip OrganoPlate devices have successfully been used to mimic intestinal, glomerular and blood-brain barrier models as they address several limitations associated with other organ-on-chip formats.^25^^,^^26^^,^^27^^,^^28^ Firstly, they contain multiple miniaturized physiological models in a multi-well format, facilitating assay standardization and are scalable for high-throughput applications. The PhaseGuide technology used enables compartments to be directly connected in absence of an artificial membrane, a configuration that is closer to the physiological setting and well-suited for permeability and transport studies.^29^ Combined with the use of a plate made of glass and polymers overcoming drug absorption issues associated with PDMS, this format is particularly advantageous for drug screening applications. Moreover, a flow is applied within micro-channels using a gravity-driven system without the need for complex pumps and tubing.^30^ In particular, co-culturing BeWo b30 and primary endothelial cells in the OrganoPlate 3-lane platform recently proved successful in replicating placental barrier functions and disease phenotypes.^31^^,^^32^ Yet these models are yet to be fully characterized and use cell lines as a surrogate for trophoblast. Implementing more physiologically relevant trophoblast alternatives and more scalable devices are necessary steps before placenta-on-chip systems can be considered as unique and reference models of the placental barrier.

The emerging use of stem cell-derived trophoblasts opened new perspectives for placental modeling. Especially, it was found that Bone Morphogenetic Protein 4 (BMP4) treatment of human induced pluripotent stem cells (hiPSC) coupled to an inhibition of basic fibroblast growth factor (FGF-2) and Activin/Nodal signaling, referred to as “BAP” treatment, specifically mediated trophoblastic differentiation.^33^^,^^34^^,^^35^^,^^36^^,^^37^ BAP-derived trophoblasts from hiPSC are known to reflect the primitive syncytium and show very little resemblance to BeWo cells, therefore showing great potential as a more physiologically relevant alternative to cancerous cell lines in placental barrier models.^38^^,^^39^ Moreover, hiPSC be propagated in long-term cultures and have the potential to recapitulate patient-specific processes by being easily derived from individuals with less ethical issues compared to human embryonic stem cells (hESC). The hiPSC-derived trophoblast model has notably proved useful to study pregnancy complications such as preeclampsia, complete hydatidiform mole or genetic disorders such as human monosomy X.^34^^,^^35^^,^^36^ Although hiPSC-derived trophoblasts are known to produce a functional primitive syncytium phenotype and have been used for studying hormone secretion and cell fusion processes, their potential to replicate and study the full range of placental barrier functions, such as implantation or placental transport, remains only partially uncovered.^33^^,^^34^^,^^35^^,^^36^^,^^39^^,^^40^ A major reason is that hiPSC-derived trophoblasts have been so far mostly restricted to conventional 2D culture settings, which are poorly amenable to transport studies because of their inability to replicate a polarized barrier with access to the apical and basal compartments.^25^^,^^26^^,^^27^^,^^28^^,^^29^^,^^30^^,^^31^

Here, we aimed at developing a novel placental barrier model combining advantages of the multi-chip, perfusable microfluidic culture with those of the hiPSC-derived trophoblasts by direct trophoblast differentiation of hiPSC within an OrganoPlate 3-lane device.

When differentiated within the microfluidic device, we found that hiPSC-derived trophoblasts rapidly self-assembled into a 3D tubular structure composed of a differentiated, invasive primitive syncytium forming a structural barrier and expressing a wide array of placental transporters. Comparative transcriptomic analysis revealed transcriptional changes on microfluidic culture, which altogether pointed out toward enhanced cell interactions with the microenvironment and surface receptor signaling. These results demonstrated that using hiPSC-derived trophoblasts within a microfluidic format provided the ability for addressing the current limitations and improving standardization of placenta-on-chip platforms on one hand, while equally improving the physiological relevance and applicability of the hiPSC-derived trophoblast model.

## Results

### Validation of trophoblast differentiation protocol in 2D culture conditions

Because the BAP differentiation protocol to generate trophoblasts has been developed in hESC by Amita et al.,^41^ only a few studies have applied it to hiPSC and experimental conditions are highly variable across experiments in terms of hiPSC origins and composition of the basal culture medium used for hiPSC maintenance and differentiation.^33^^,^^34^^,^^35^^,^^36^^,^^37^^,^^42^ Here, we used ChiPS4, a hiPSC cell line derived from primary human dermal fibroblasts, and a non-conditioned mTeSR1 medium for cell maintenance and BAP differentiation, a combination that has not been reported for trophoblast differentiation elsewhere. Therefore, it was important to first validate the trophoblast differentiation protocol by assessing morphological features and measuring molecular markers throughout 6 days of BAP exposure. These molecular markers were selected as they have been extensively used in the literature to validate hiPSC-derived trophoblast differentiation, and include a mix of pan-trophoblast, CTB, EVT, and STB specific markers.^33^^,^^34^^,^^41^^,^^43^

**Figure 1:**
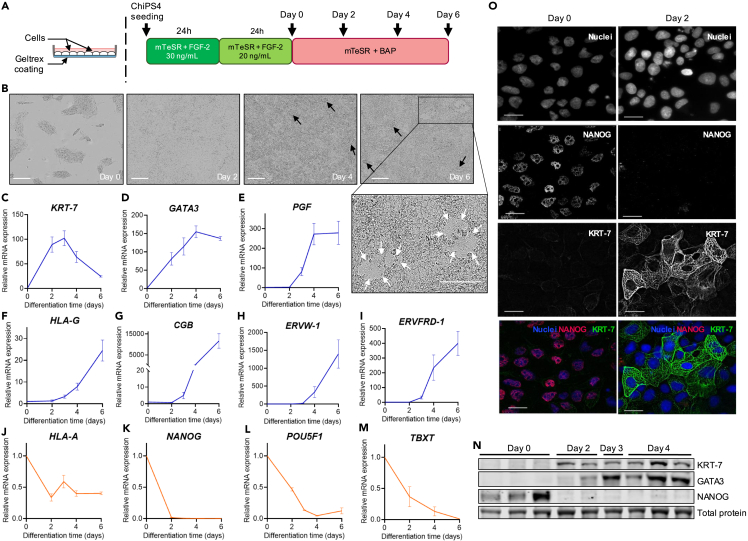
Validation of trophoblast differentiation protocol in 2D culture conditions (A) Schematic diagram of experimental setup (left), culture media and timeline (right) used for ChiPS4 differentiation into trophoblasts. BAP = BMP4 10 ng/mL + A83-01 1 μM + PD173074 0.1μM. (B) Representative phase-contrast images of ChiPS4 exposed to mTeSR + BAP differentiation media at Day 0, 2, 4 and 6. Black arrows indicate cell aggregates growing vertically and shedding from the cell layer and white arrows indicate areas resembling fused syncytium. Scale bar, 200 μm. (C–M) Expression levels of *KRT-7, GATA3, PGF, HLA-G, CGB, ERVW-1, ERVFRD-1, HLA-A, NANOG, POU5F1* and *TBXT* after 2, 4 and 6 days of BAP exposure compared to day 0 levels assayed by RT-qPCR and normalized to *GAPDH*. Relative mRNA expression is shown as mean ± SEM (n = 3). Blue lines = positive trophoblast markers; orange lines = negative trophoblast markers. (N) Representative western blot gels showing protein levels of KRT-7, GATA3 and NANOG at day 0, 2, 3 and 4 of BAP differentiation. (O) Representative images of ChiPS4 fixed before (Day 0) and after 2 days of BAP exposure and stained with DAPI (blue), NANOG (red) and KRT-7 (green). Scale bar, 10 μm.

After seeding in mTeSR medium containing 30 ng/mL FGF-2, ChiPS4 were cultured in reduced FGF-2 medium for 24 h before being exposed to the final differentiation medium lacking FGF-2 and supplied with BAP factors for 6 days (Figure 1A). Phase-contrast images of the BAP-treated hiPSC showed a rapid loss of pluripotent cell phenotype within 48 h and the progressive transition to a flattened epithelial-like morphology (Figure 1B). BAP-exposed cells formed a confluent monolayer after 48 h, after which they quickly became overconfluent as seen by the formation of cell aggregates detaching from the layer from day 3 as previously reported in hESC-derived trophoblasts (Figure 1B).^43^

A combination of mRNA and protein measurements showed a rapid down-regulation of hiPSC pluripotency markers *NANOG* and *POU5F1* as soon as 2 days after BAP exposure, parallel to a gradual expression of pan-trophoblast markers cytokeratin 7 (*KRT-7*), GATA-binding protein 3 (*GATA3*) and placental growth factor (*PGF*) throughout 6 days of BAP exposure (Figures 1C–1E, 1K–1L, and 1N–1O). In addition,^43^ the mesoderm-specific marker Brachyury (*TBXT*) was continuously down-regulated and reached undetectable levels at day 6 (Figure 1M).

The increasing expression of syncytin-2 (*ERVFRD-1*) reflected the presence of CTB within the cell population.^44^ RT-qPCR results showed acquisition of a human leukocyte antigen (HLA) class I molecule profile that is unique to EVT, characterized by up-regulation of HLA-G (*HLA-G*) and down-regulation of HLA-A (*HLA-A*) antigens^43^ (Figures 1F and 1J). The tremendous levels of placental hormone β-hCG (*CGB*) expressed by the cell population from day 4 was in accordance with previous BAP differentiation studies using hiPSC and hESC and reflected the presence of functional STB in the trophoblast population (Figure 1G).^39^^,^^41^^,^^42^ The occurrence of spontaneous syncytialization events was further supported by high levels of the fusogenic gene syncytin-1 (*ERVW-1*) and the presence of discontinuous fused areas resembling syncytium scattered across the cell layer in accordance with previous reports (Figures 1B and 1H–1I).^33^^,^^41^^,^^45^CTB, STB and EVT-specific markers were detected after 3 days and were increasingly expressed up to 6 days, the latest time point measured, with STB-specific markers *CGB* and *ERVW-1* expressed to a much higher extent compared to EVT-specific marker *HLA-G* (Figures 1F–1I).

Altogether, these results confirmed that the modified BAP protocol employed herein was efficient at committing ChiPS4 cells to the trophoblastic lineage and generated a mixed population of CTB, STB and EVT over 6 days of differentiation.

### Generation of hiPSC-derived trophoblasts in a 3D microfluidic device

**Figure 2:**
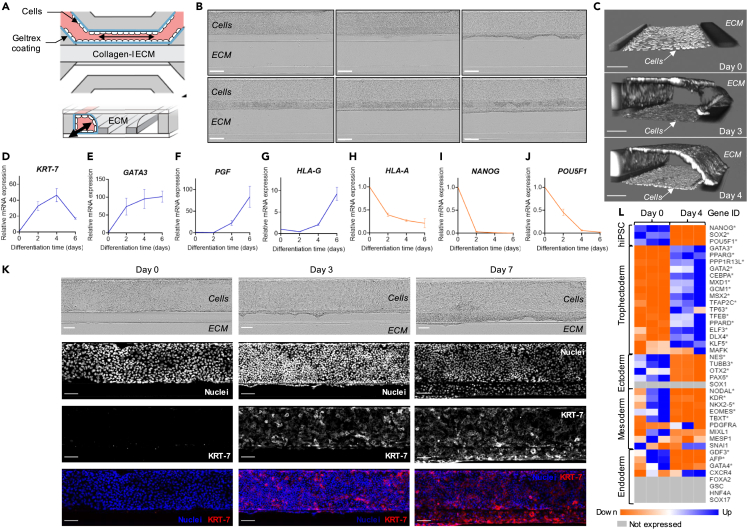
Generation of hiPSC-derived trophoblasts in a 3D microfluidic device (A) Schematic diagram of experimental setup used for ChiPS4 differentiation into trophoblasts in the OrganoPlate 3-lane device (Adapted from Duinen et al.^46^). Black arrows indicate the bidirectional flow. (B) Representative phase-contrast images of ChiPS4 differentiating in the OrganoPlate 3-lane device between day 0 and day 6 of BAP treatment. Scale bar, 200 μm. (C) 3D reconstructions of confocal images of ChiPS4 stained with nuclei marker Hoechst at day 0, 3 and 4 of BAP treatment. Scale bar, 100 μm. (D–J) Expression levels of *KRT-7, GATA3, PGF, HLA-G, HLA-A, NANOG* and *POU5F1* after 2, 4 and 6 days of BAP exposure compared to day 0 levels assayed by RT-qPCR and normalized to *GAPDH*. Relative mRNA expression is shown as mean ± SEM (n = 3). Blue lines = positive trophoblast markers; orange lines = negative trophoblast markers. (K) Representative images of ChiPS4 fixed before (Day 0), after 3 and 7 days of BAP treatment and stained with nuclei marker Hoechst (blue) and KRT-7 (red). Scale bar, 100 μm. (L) Heatmap showing relative expression of molecular markers for hiPSC, trophectoderm, ectoderm, mesoderm and endoderm lineages selected from the literature^47^^,^^48^^,^^49^^,^^50^^,^^51^^,^^52^ before (Day 0) and after 4 days of BAP treatment sorted by adjusted p value (padj). Genes that differed significantly in expression (log2FoldChange>1 and padj<0.05) between day 0 and 4 are marked with ∗. Gray rectangles show genes with an average read count <10. See also Table S1.

To create a hiPSC-derived placenta-on-a-chip platform, we seeded undifferentiated ChiPS4 cells in an OrganoPlate 3-lane device and applied a similar BAP differentiation protocol as detailed above. The experimental setup in the microfluidic chip is depicted in Figure 2A detailed description can be found in the STAR methods section. Briefly, ChiPS4 cells were seeded against a collagen-1 ECM and trophoblast differentiation was driven by exposing ChiPS4 to the same succession of media as used in the 2D culture setting while maintaining a continuous biphasic flow in the top lane containing the cells. Cells successfully attached to the ECM and proliferated, progressively covering the entire top channel walls and forming a hollow tube structure after 4 days of BAP exposure (Figures 2B and 2C). From day 4 to day 6, cells formed a uniform front at the apical side in contact with the ECM in the middle lane. The hiPSC-differentiated cells displayed an invasive behavior by entering the middle ECM compartment (Figure 2B).

The expression timeline of commonly used positive and negative trophoblast markers throughout 6 days of BAP treatment showed comparable temporal expression to those observed in the 2D setting with pluripotency markers *NANOG* and *POU5F1* as well as somatic cell marker *HLA-A* down-regulated, and trophoblast markers *KRT-7, GATA3, PGF*, and *HLA-G* up-regulated on BAP treatment (Figures 2D–2J). In addition, KRT-7 immunostaining in the tubular structure formed by the cells confirmed gradual increase in protein expression lasting up to 7 days after starting BAP treatment (Figure 2K).

RNA sequencing (RNA-seq) analysis was performed on RNA from independent samples of ChiPS4 differentiating in the OrganoPlate before (Day 0) and after 4 days (Day 4) of BAP treatment. A differential gene expression analysis was performed between day 0 and day 4 samples to decipher transcriptomic changes after 4 days of BAP treatment.

To further confirm the trophoblastic identity and specificity of the BAP-derived population obtained in the microfluidic platform, we selected markers of trophectoderm and other lineage commitments from the literature and compared their expression before and after 4 days of BAP treatment.^47^^,^^48^^,^^53^^,^^54^^,^^55^ All three transcription factors (*POU5F1*, *SOX2*, and *NANOG*), making up the core transcriptional pluripotency regulatory network, showed a significant and strong down-regulation confirming unidirectional cell differentiation (Figures 2I–2J, 2L, and Table S1).^47^ Given the limited number of genes that are truly restricted to the human trophectoderm, we measured expression levels of the 16 transcription factors identified by Bai et al. as making up the core transcriptional network involved in establishing and maintaining the trophoblast lineage.^48^ Out of these 16 transcription factors, 15 were found to be significantly up-regulated (Figures 2E, 2L, and Table S1). Those included *GCM1*, a transcription factor uniquely expressed in the trophectoderm and mature placenta, and *PPARG,* both necessary for placental development in mice.^48^^,^^53^^,^^54^^,^^55^ The only transcription factor of the network that was not found to be up-regulated, *MAFK,* showed strong and constant expression between day 0 and day 4 indicating that the gene was already expressed before BAP treatment and maintained throughout the 4 days of differentiation (Table S1).

Most commonly used representative markers of the mesoderm, endoderm and ectoderm lineages were then selected from the literature.^39^^,^^49^^,^^50^^,^^51^^,^^52^ After 4 days of BAP treatment, all markers tested were either found significantly down-regulated, unchanged or not expressed at a biologically meaningful level (Figure 2L, and Table S1).

The strong and concomitant expression of all transcription factors making up the trophectoderm core transcriptional circuitry combined with the absence of hiPSC as well as mesoderm, endoderm and ectoderm lineage markers brought evidence that BAP treatment drove a unidirectional and specific trophoblast differentiation in the microfluidic platform.

### On-chip trophoblast differentiation of hiPSC produces a functional syncytium

Recent transcriptomic data from single-cell studies or primary cells derived from human placenta identified signature genes that are highly expressed on trophoblast differentiation into CTB, STB or EVT trajectories.^39^^,^^56^^,^^57^^,^^58^ Based on these findings, we compiled a list of 39, 46, and 30 markers indicative of CTB, STB or EVT differentiation respectively and examined changes in their expression after 4 days of BAP differentiation to evaluate the presence of different trophoblast subtypes in the resulting cell population.

**Figure 3:**
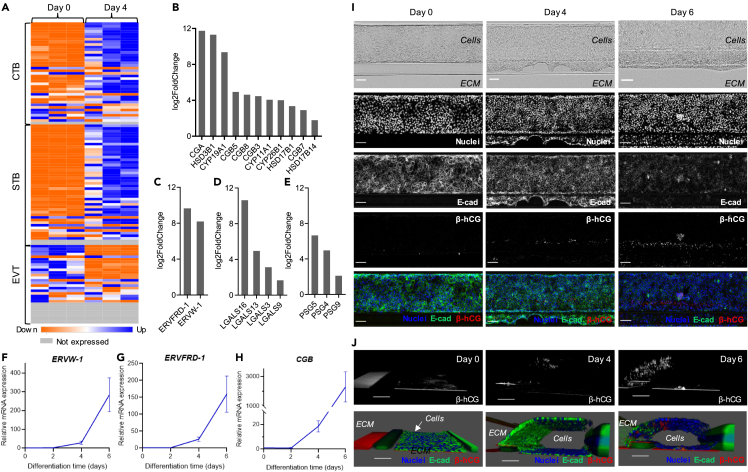
On-chip trophoblast differentiation of hiPSC produces a functional syncytium (A) Heatmap showing relative expression of CTB, STB and EVT-associated genes selected from the literature^39^^,^^56^^,^^57^^,^^58^ before (Day 0) and after 4 days of BAP treatment sorted by padj. See also Table S2 and Figure S3. (B–E) Histograms showing log2FoldChange of selected STB markers related to hormone synthesis (B), cell fusion (C), L-galectin production (D) or pregnancy-specific β1 glycoprotein production (E) showing significant up-regulation after 4 days of BAP treatment in the microfluidic chip. See also Table S3. (F–H) Expression levels of *ERVW-1*, *ERVFRD-1* and *CGB* after 2, 4 and 6 days of BAP exposure compared to day 0 levels assayed by RT-qPCR and normalized to *GAPDH*. Relative mRNA expression is shown as mean ± SEM (n = 3). (I) Representative images of ChiPS4 fixed before (Day 0), after 4 and 6 days of BAP treatment and stained with nuclei marker Hoechst (blue), E-cadherin (green) and β-hCG (red). Scale bar, 100 μm. (J) 3D reconstructions of confocal images taken of the same chips displayed in C showing β-hCG signal alone (top) or Hoechst (blue), E-cadherin (green) and β-hCG (red) merged (bottom). Scale bar, 100 μm.

RNA-seq and differential gene expression analysis revealed strong up-regulation of most STB markers after 4 days of BAP treatment (Figures 3A, S3, and Table S2). Those included α and β subunits of hCG (*CGA, CGB3, CGB5, CGB7* and *CGB8*), enzymes involved in estrogen and progesterone biosynthesis (*HSD3B1*, *HSD17B1*, *CYP11A1*, and *CYP19A1*) as well as proteins from the pregnancy-specific glycoprotein (PSG) and L-galectin (LGALS) families that are abundantly expressed by STB (*PSG4* and *LGALS16*), indicating the presence of fully differentiated syncytium (Figures 3B, 3D–3E, Tables S2 and S3).^39^^,^^59^^,^^60^ In addition, several other genes from the HSD, CYP, PSG, and LGALS families that have been identified in syncytialized primary human trophoblasts were detected at day 4 (Figures 3B, 3D–3E, and Table S3).^39^ In contrast, genes encoding placental lactogen (*CSH1* and *CSH2*) and placental alkaline phosphatase (*ALPP*), associated with term placentas, were not expressed after 4 days of BAP differentiation (Table S3).^38^^,^^39^

The fusogenic placenta-specific envelope proteins syncytin-1 and syncytin-2 (*ERVW-1* and *ERVFRD-1*) were strongly upregulated, reflecting the presence of CTB fusing into STB (Figures 3C, 3F–3G, Tables S2 and S3).^44^ Besides *ERVFRD-1*, a panel of CTB markers were upregulated after 4 days of BAP treatment (Figures 3A, S3, and Table S2). Among them were several stemness related genes, such as *TFAP2C*, *CDX2* and *ELF5,* further supporting the presence of an undifferentiated CTB population in the model (Figures 3A, S3, and Table S2).^56^^,^^58^

Although some EVT-specific markers such as HLA-G were upregulated after 4 days, many genes related to migration or epithelial-mesenchymal transition during EVT differentiation were either not expressed, not significantly changed or down-regulated after 4 days of BAP treatment (Figures 3A, S3, and Table S2).

Temporal changes in *ERVW-1*, *ERVFRD-1* and *CGB* gene expression throughout 6 days of BAP treatment was further assessed by RT-qPCR. All four genes showed a similar timeline of expression with minimal expression after 2 days, moderate induction after 4 days and maximal mRNA levels after 6 days of BAP treatment, reaching almost 300-fold increase for *ERVW-1* and more than 2000-fold for *CGB* (Figures 3F–3H).

To localize hormone production and cell fusion events directly within the tubular structure, placental hormone β-hCG and plasma membrane protein E-cadherin were detected by immunostaining. β-hCG was gradually expressed between day 4 and day 6, and showed an interesting geographical pattern of expression as β-hCG signal was mostly restricted to a defined outer layer in close proximity to the ECM (Figures 3I and 3J). This high β-hCG production area matched with local loss of plasma membrane protein E-cadherin, a marker commonly used to identify fusing trophoblasts during syncytium formation (Figures 3I and 3J).^6^^,^^61^ 3D reconstructions of confocal images confirmed progressive β-hCG induction against the ECM matrix, a signal distinct from a constant red signal observed on the basal side that was attributed to auto-fluorescence of the plate (Figure 3J).

Together, these data demonstrated that the hiPSC-derived mixed trophoblast population derived in the microfluidic platform contains fully differentiated, functional STB capable of fusogenic and endocrine activities.

### Evaluation of the hiPSC-derived placenta-on-chip model to serve for placental barrier transport studies

**Figure 4:**
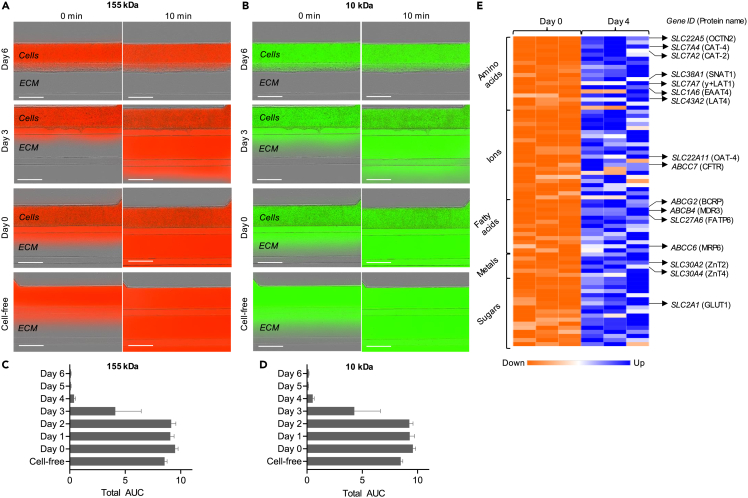
Evaluation of the hiPSC-derived placenta-on-chip model to serve for placental barrier transport studies (A and B) Representative images showing fluorescence signal 10 min after adding 155 kDa TRITC-Dextran (A) or 10 kDa FITC-Dextran (B) compounds in the top channel of day 0, 3 and 6 cultures compared to a cell-free channel. Scale bar, 400 μm. See also Figure S1. (C and D) Total area under the curve (AUC) quantified from ratio values of 155 kDa TRITC-Dextran (C) or 10 kDa FITC-Dextran (D) fluorescence signal in the ECM channel to signal in the cell compartment calculated daily from independent experiments (n = 3). Values are shown as mean ± SEM. (E) Heatmap showing relative expression of all ABC and SLC transporters found significantly upregulated after 4 days of BAP treatment, grouped by main physiological substrate category and sorted by padj. Gene ID and protein names of known placental transporters are indicated on the right. See also Table S4.

We evaluated the structural integrity of the hiPSC-derived trophoblast tubular structure obtained in the microfluidic platform by assessing its permeability to compounds of various molecular sizes. After 155 kDa TRITC-Dextran or 10 kDa FITC-Dextran fluorescent tracers were introduced into the top micro-channel compartment containing the trophoblasts, the resultant fluorescence intensity in the ECM gel compartment was monitored over 10 min to focus on paracellular transport and normalised to the fluorescence signal present in the top channel as a measurement of tracer leakage. Up to day 2, both fluorescent compounds leaked from the cell vessel in all chips measured (Figures 4A–4D and S1). Leak-tight properties were obtained after 3 days only for some chips, although that was not always the case (Figures 4A–4D and S1). Starting from day 4 and up to day 6, the complete absence of fluorescent signal in the gel compartment after 10 min attested that the cell structure formed a leak-tight barrier to both 155 kDa and 10 kDa compounds (Figures 4A–4D and S1).

To assess the barrier’s potential to serve as a model for placental transport, we screened all ATP-binding cassette (ABC) and solute carrier (SLC) transporters showing significant up-regulation after 4 days of BAP treatment. RNA-seq and differential gene expression analysis showed significant up-regulation of 10 ABC transporters and 66 SLC transporters, involved in trafficking of diverse physiological compounds such as glucose, amino acids, fatty acids, metals and ions (Table S4). Some well-studied placental transporters up-regulated in our barrier model included the facilitative glucose transporter GLUT1, considered the primary placental transporter of glucose and required for its directional net transport from the mother to the fetus as well as multiple known placental amino acid transporters belonging to accumulative, exchange and facilitated transporter categories required to transport the full range of amino acids to the fetus^62^^,^^63^^,^^64^ (Figure 4E and Table S4). Other placental transporters included FATP6 involved in transplacental transport of fatty acids, ZnT2 and ZnT4 involved in cadmium transport from mother to fetus, as well as CFTR known to mediate chloride uptake in the placenta (Figure 4E and Table S4).^65^^,^^66^^,^^67^

Importantly, multiple transporters that have been reported to function as drug transporters in the human placenta were found upregulated in our model. Those included several efflux drug transporters such as MDR3, MRP6 or the breast cancer resistance protein (BCRP) that is widely expressed on the apical membrane of the STB and known as a major transporter responsible for drug resistance in the placenta (Figure 4E and Table S4).^68^^,^^69^^,^^70^^,^^71^ Other drug transporters upregulated in our model included the placental organic anion transporter OAT-4 and organic cation/carnitine transporter OCTN2 (Figure 4E and Table S4).^72^^,^^73^^,^^74^

### Microfluidic culture alters the transcriptomic profile of hiPSC-derived trophoblasts

For further analysis, we evaluated the global effects of microfluidic culture conditions on the transcriptomic profile of hiPSC-derived trophoblasts by comparing gene expression patterns of hiPSC-derived trophoblasts differentiated in the microfluidic device against hiPSC-derived trophoblasts differentiated in a conventional 2D culture plate. RNA was isolated from each group at day 4 of BAP treatment and used for RNA-seq. A differential gene expression analysis was performed to identify differentially expressed genes between hiPSC-derived trophoblasts differentiated in microfluidic or 2D culture conditions.

**Figure 5:**
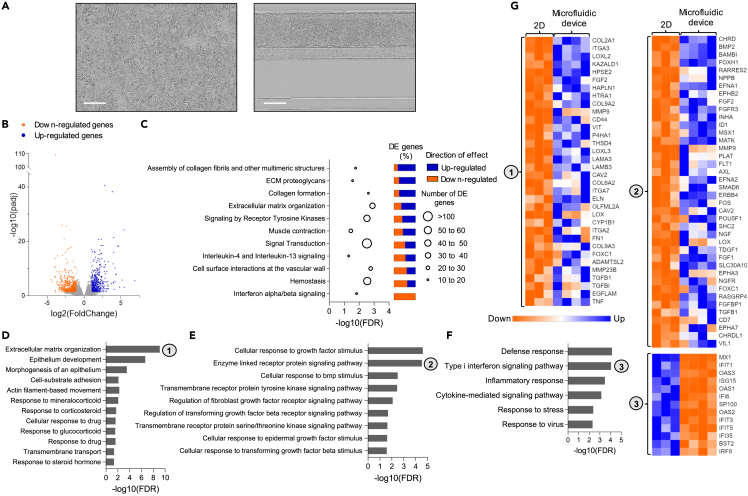
Microfluidic culture alters the transcriptomic profile of hiPSC-derived trophoblasts (A) Representative phase-contrast images showing hiPSC-derived trophoblasts in conventional 2D culture setting (left) or in the OrganoPlate microfluidic device (right) after 4 days of BAP treatment. Scale bar, 200 μm. (B) Global transcription changes in Day 4 cells differentiated in the microfluidic chip vs. 2D plates visualized by a volcano plot. Genes with a padj<0.05 and log2FoldChange<−1 are indicated by orange dots and represent down-regulated genes. Genes with padj<0.05 and log2FoldChange>1 are indicated by blue dots and represent up-regulated genes. See also Table S5. (C) Graph showing Reactome pathways enriched in the list of 1,019 differentially expressed genes between Day 4 microfluidic vs. 2D cultures with associated false discovery rates (FDR). The number of differentially expressed genes per pathway are depicted by circle size. For each gene set, the relative amounts of down-regulated (red) and up-regulated (green) genes are indicated on the right. See also Table S6. (D–F) Graphs showing selected GO terms representing biological processes enriched specifically in up-regulated genes (D-E) or in down-regulated genes (F) with associated FDR. See also Table S6. (G) Heatmaps showing relative expression of all genes belonging to GO terms *Extracellular matrix organization* (1), *Enzyme linked receptor protein signaling pathway* (2) and T*ype I interferon signaling pathway* (3) that were found significantly changed between Day 4 microfluidic vs. 2D cultures, sorted by padj. See also Table S7.

Out of the 17,213 genes measured, a total of 1,019 genes (5.92%) were differentially expressed between both culture conditions, among which 462 were upregulated and 557 were downregulated in the microfluidic against 2D culture (Figure 5B and Table S5). Most significant up-regulated genes included Stanniocalcin-1 (*STC1*, log2FC = 2.80, padj = 2.62 × 10^−41^), a glycoprotein hormone secreted by first trimester STB, carbonic anhydrase 3 (*CA3*, log2FC = 3.84, padj = 5.67 × 10^−39^) and NK2 Homebox 6 transcription factor (*NKX2-6*, log2FC = 5.42, padj = 6.03 × 10^−25^).^75^ Most significant down-regulated genes included MX dynamin like GTPase 1 (*MX1,* log2FC = −3.85, padj = 7.91 × 10^−110^), Interferon induced protein with tetratricopeptide repeats 1 (*IFIT1,* log2FC = −2.99, padj = 9.92 × 10^−27^) and 2′-5′-oligoadenylate synthetase 3 (*OAS3,* log2FC = −2.39, padj = 2.92 x 10^−26^), all interferon-induced proteins.

To obtain a general overview of the cell components and biological pathways altered by the microfluidic culture, we determined whether there was an overrepresentation of total differentially expressed genes in terms that are part of the Gene Ontology (GO) knowledgebase or the Reactome pathway database. Overrepresentation analysis showed enrichment of differentially expressed genes in 30 GO terms related to cellular components that were all linked to cell membrane, ECM or cytoskeleton dynamics (Table S6). Pathway enrichment analysis showed enrichment of differentially expressed genes in 11 Reactome pathways (Figure 5C, Table S6). Among enriched pathways were many linked to ECM organization such as such as *Assembly of collagen fibrils and other multimeric structures*, *ECM proteoglycans, Collagen formation* and *ECM organization* in accordance with the GO term analysis. Most differentially expressed genes were up-regulated in those categories, indicating a global increase in the activation of processes mediating ECM rearrangements in the microfluidic device.

Several other enriched biological pathways were linked to surface receptors and signal transduction, such as *Signaling by receptor tyrosine kinases, Signal Transduction, Interleukin-4 and Interleukin-13 signaling*, *Cell surface interactions at the vascular wall* and *Interferon alpha/beta signaling,* indicating that receptor signal transduction was affected by the microfluidic culture (Figure 5C and Table S6). Although both up-regulated and down-regulated genes were represented in most of these categories, it was striking to see that all genes included in the *Interferon alpha/beta signaling* pathway category were down-regulated on differentiation in the microfluidic device, reflecting that microfluidic culture globally decreased interferon alpha/beta signaling compared to the 2D culture setting.

To obtain further insights into the biological significance of transcriptomic changes, we performed an overrepresentation analysis on the lists of up-regulated and down-regulated genes separately and distinguished the GO terms enriched specifically in each list (Table S6). *ECM organization* (FDR = 8.16 x 10^−10^) was one of the top biological processes enriched specifically in up-regulated genes, further supporting that microfluidic culture increased activation of cell-ECM interactions in accordance with the previous overrepresentation analysis (Figure 5D). Up-regulated genes in this category included multiple ECM components, adhesion receptors, enzymes regulating ECM assembly or degradation as well as growth factors (Figure 5G and Table S7). Other biological processes of interest enriched specifically in up-regulated genes were related to epithelium development, response to drug and transport functions suggesting that the microfluidic setting boosted epithelium differentiation and the placental barrier functionality (Figure 5D).

Given previous overrepresentation analysis on total differentially expressed genes pointed toward a global alteration in surface receptors and signal transduction, we then focused on related biological processes enriched specifically in up-regulated or down-regulated gene lists. Biological processes enriched specifically in up-regulated genes included many GO terms related to cellular response to growth factors and associated enzyme-linked surface receptor signaling, indicating enhanced response to growth factor stimulus on microfluidic culture (Figure 5E). Of interest, up-regulated tyrosine kinase receptors included several well-known players in pregnancy disorders like preeclampsia, such as AXL, ERBB4 or FLT1 (Figure 5G and Table S7).^76^^,^^77^^,^^78^ On the other hand, *Type I interferon signaling pathway* was one of the top pathways enriched specifically in down-regulated genes and analysis of down-regulated gene protein-protein interaction network on STRING revealed a clear local cluster involved in interferon alpha/beta signaling (Figures 5F–5G and S2). This further confirmed that interferon signaling was specifically decreased on microfluidic culture compared to the 2D conditions in accordance with the previous overrepresentation analysis of total differentially expressed genes. More generally, GO terms enriched exclusively in down-regulated genes included many terms linked to stress and inflammatory response (Figures 5F and 5G).

## Discussion

hiPSC-derived trophoblast models of the early syncytium constitute an important alternative to cell lines in placenta-on-chip systems in term of physiological relevance, potential for patient-specific models and to serve as a replacement for animal models. Yet, the potential of hiPSC-derived trophoblasts to replicate a functional placental barrier when cultured in a microfluidic platform has to our knowledge not been investigated before. In the current study, we designed and characterized a new approach to modeling the placental barrier *in vitro*, where hiPSC are cultured and differentiated into trophoblasts in the top channel of a multi-well microfluidic platform.

After validating that the BAP protocol was efficient at deriving trophoblasts from ChiPS4, l, we implemented the same differentiation protocol directly within an OrganoPlate 3-lane microfluidic platform and confirmed the generation of a mixed trophoblast population of CTB, STB, and EVT. Although a vast majority of STB markers measured showed robust upregulation on BAP treatment, the fact that only a few EVT markers were induced suggested that STB was the major terminally differentiated trophoblast subtype present in the model, probably co-existing with a smaller, or incompletely differentiated, EVT subpopulation. This was expected as the BAP protocol is known to favor STB over EVT differentiation when performed under 20% O_2_ conditions.^33^^,^^35^^,^^41^

After 4 days of BAP treatment, hiPSC-derived trophoblasts self-arranged into a 3D, hollow tubular structure showing multiple features that are typical of a functional primitive syncytium. These included invasive behavior, expression of syncytin-1 and 2 reflecting the presence of CTB fusing into STB, endocrine activities and expression of placental transporters.^44^ Moreover, the strong and stable structural integrity of the tubular structure indicated the ability of the epithelial layer to form and maintain a physical barrier between two distinct environments, the ECM on the apical side and the flowing medium on the basal side, which is a strong indicator of successful syncytialization of the trophoblast epithelium and its differentiation into a functionally polarized barrier.^15^^,^^79^

One of the most distinctive features brought by the microfluidic environment was the structural organization of differentiating hiPSC compared to the conventional 2D culture. Indeed, a defined area in close proximity to the ECM appeared as a preferential site for cell fusion and β-hCG production and therefore likely constituted a syncytialized layer progressively differentiating from CTB present on the basal side. This spatiotemporal control of syncytialization resembles the *in vivo* situation in which the external STB barrier forms by progressive fusion of underlying CTB.^1^ We therefore hypothesized that this asymmetric organization reflects the maternal-fetal interface formation, with the outside of the tubule in contact with the ECM representing the maternal-facing syncytium, and the basal side of the tubule the fetal compartment containing single CTB. In contrast, syncytium-like areas occurred discontinuously within the trophoblast monolayer when differentiated in a conventional 2D culture setting, as shown here and in previous reports, which is less representative of human placental development.^33^^,^^41^^,^^45^

The fact that the microfluidic culture alone allowed this self-organization into defined layers is of particular interest given that hiPSC-trophoblasts cultured in a 3D gel matrix require other cell types to adopt similar spatial organisation.^80^ Given the importance of mechanical cues in controlling the syncytialization process and more specifically cell fusion and hormone release, it is not surprising that the direct ECM contact at the apical side, continuous shear stress, and distinct surface-to-volume ratios present in the microfluidic system are promoting hiPSC differentiation into STB.^81^ Cell responses to biophysical cues imposed by the microfluidic format were further reflected in comparative transcriptomics studies that highlighted ECM organization, cell-substrate adhesion and cell dynamics as primary biological processes enhanced on microfluidic culture. This was expected as increased expression of genes related to ECM organization, cell adhesion and migration has been reported in several other cell types cultured in 3D vs. 2D culture settings, and highlights a critical and overlooked limitation of the current standard 2D cell studies.^82^^,^^83^ Given the growing interest in the role of mechanosensing in placental physiology and diseases, integrating microenvironment interactions increases the physiological relevance of hiPSC-derived trophoblast models and opens interesting perspectives for research applications.^81^^,^^84^

Enrichment for processes linked to ECM organization, cell dynamics and interleukin signaling in the microfluidic device were in accordance with the invasive behavior of the hiPSC-derived trophoblasts observed from day 4. These invasive abilities, coupled with an absence of late pregnancy markers, are coherent with the dominant hypothesis that BAP-derived trophoblasts represent primitive syncytium and strongly suggest that the model developed here replicates the early stages of syncytium formation happening during the implantation phase.^38^^,^^39^^,^^85^ This opens interesting perspectives for placental research studying pathological mechanisms that are linked to deficient implantation during early pregnancy, such as recurrent miscarriage.^86^

Together with cell-ECM interactions, microfluidic culture boosted expression of genes linked to epithelium development and surface receptor signaling. Altogether, these transcriptomics results further supported that the 3D structure formed in the microfluidic device provides a closer approximation to the human placenta as cell communication, cell surface receptor signaling and cell adhesion are central pathways related to the cellular organization of the placenta’s structure that are enriched in early placental development.^87^ Of interest, among surface receptors more expressed in the microfluidic model were many central players in pregnancy complications such as FLT1, AXL, INHA, STC1 and HTRA1, supporting its use for disease modeling.^76^^,^^77^^,^^88^^,^^89^^,^^90^

Importantly, differentiating hiPSC into a microfluidic platform provides a good basis to extend the range of applications of the hiPSC-derived STB model with interesting perspectives into transport assays. Although expression of transporters has already been reported in BAP-treated hESC, the precise transporter expression profile and potential applicability of the hiPSC-derived trophoblast model for functional transport assays have been poorly documented because of the inability of conventional 2D cultures to replicate the placental barrier structure.^39^ Here, an interesting benefit brought by the microfluidic setting is the structural arrangement of hiPSC-derived trophoblasts into a 3D perfused tubular structure in direct contact with an ECM, a system that is particularly well-suited and has been successfully used for transport studies.^25^^,^^26^^,^^27^^,^^28^ We proceeded to screen all ABC and SLC transporters expressed at day 4 in our system to evaluate the model applicability for transport studies. We found a total of 76 transporters expressed, including multiple known placental transporters. The expression of GLUT1 in our model, established as the primary player in maternal-to-fetal transport of glucose, shows great potential for future analysis of glucose transfer which is the typical model substrate used to evaluate placental barrier models in term of strength, reactiveness and fluid flow in comparison with the perfused *ex vivo* human placenta.^15^^,^^16^^,^^19^^,^^32^^,^^62^ Among transporters expressed was also BCRP, one of the most important and abundant efflux transporters in the placenta responsible for drug resistance in pregnancy, which substrates include a wide range of chemotherapeutics, antiviral medication, antibiotics, calcium channel blockers, carcinogens and flavonoids and that was not expressed in other hiPSC-derived trophoblast models differentiated in a transwell setting.^91^^,^^92^ The presence of BCRP along 5 other reported placental drug transporters in our model supports its use to study drug transport across the placental barrier during pregnancy, a major application of placental barrier models given the pressing need for improvement of drug safety profiling for clinical trials in pregnancy, highlighted by the disastrous result of the STRIDER trial.^10^ In addition, our screening highlighted expression of other transporters involved in trafficking of amino acids, fatty acids or metals, which are essential characteristics when mimicking the human placenta physiology and for which testing is still lacking in current placenta-on-chip models.^11^ The enrichment for many processes linked to transmembrane transport and response to drug in the microfluidic environment further supported the pertinence of the microfluidic format described herein for functional placental barrier transport and drug toxicity studies.

Culturing hiPSC-derived trophoblasts in a microfluidic culture also demonstrated potential to maintain cultures for a longer time frame compared to the 2D setting. In traditional 2D settings, cells became quickly overconfluent and started shedding from the monolayer as soon as 4 days after starting the BAP treatment. This issue, also encountered by other groups including when using mesh cultures, severely complicates functional studies.^43^^,^^92^ Microfluidic culture supported cell growth, proliferation and STB differentiation over at least 7 days of BAP exposure. In addition, a global decrease in stress and defense responses parallel to increased response to growth factors suggested that cells were healthier and proliferated better in the microfluidic device compared to the 2D culture plate, although we did not directly test cell viability. Strikingly, the microfluidic format led to a global decrease in interferon-induced proteins. In view of the known antiviral properties of interferon signaling and its role in the blockade of syncytin-mediated trophoblast fusion, a global reduction in interferon signaling may reflect a facilitated syncytialization process in the microfluidic device compared to the 2D culture.^93^^,^^94^

In light of the limitations associated with current placental barrier models, implementing hiPSC into an OrganoPlate platform appears advantageous in term of physiological relevance, practicality and potential applications. The ability of hiPSC-derived trophoblasts to spontaneously form a syncytium resembles the behavior of primary CTB cultures *in vitro.*^95^ This spontaneous fusion is a unique feature compared to immortalized cell lines commonly used in placental barrier models, as well as other hiPSC-derived STB models using experimental conditions that differed from our study, all requiring a chemical treatment to syncytialise.^13^^,^^15^^,^^42^^,^^92^ By better replicating the natural structural arrangement of the placenta, self-organizing hiPSC-derived trophoblasts represent a more physiologically relevant alternative to immortalized cell lines and might serve to provide additional cues on the mechanisms regulating spatiotemporal differentiation into the syncytial layer during early stages of placental development. Moreover, a spontaneous syncytium formation provides benefits in term of experimental practicality, given chemical induction is often not sufficient to achieve syncytialization of the entire barrier and raises issues when exposing other cell types in co-cultures.^13^^,^^15^ Finally, the use of the ordinary and commercially available hiPSC cell lines ChiPS4, combined with a readily accessible microfluidic tool compatible with standard lab instruments for analysis, makes this placental barrier model easily implementable in any lab setup and very practical for other researchers to adopt.

Our findings demonstrated the feasibility of using hiPSC-derived trophoblasts to make up a functional syncytium layer in placenta-on-chip systems. In addition to providing insights into the influence of a microfluidic environment on hiPSC-derived trophoblast models, this study constitutes an important resource that can be further explored to improve placental barrier models.

### Limitations of the study

To validate the applicability of our model for transport studies, it would be relevant to evaluate the spatial expression pattern of transporters and to monitor uptake of reference compounds to confirm that the barrier replicates the transport functions and semi-permeability of a physiological placental barrier.

Although the maintenance of barrier integrity over several days strongly supported a functional polarization of the placental barrier, further studies would be needed to get some direct evidence of polarization. Those may include localizing specific markers associated with apical or basolateral domains, or using scanning electron microscopy to check for apical microvilli formation as seen in other placenta-on-chip models using BeWo cells.^12^^,^^13^^,^^14^

In this study, we focused on characterizing the model over 7 days of differentiation to provide a direct comparison with 2D differentiation and reliable data for the influence of a microfluidic environment on hiPSC-based trophoblast differentiation. However, confirming that a functional syncytial barrier can be maintained on a longer time frame may be useful to develop relevant platforms for long-term research and therapeutic development.

Although this study explored the feasibility of using differentiated hiPSC as the trophoblast component of a placental barrier model, including other cell types that are part of the placental villi, such as endothelial cells or fibroblasts, will be useful to replicate the full placental barrier complexity and integrate cross-talks that were shown to contribute substantially to features such as barrier integrity, permeability and glucose transport.^6^^,^^15^^,^^92^

In parallel to the STB markers used in that study, further immunostaining experiments using CTB-specific markers would be needed to confirm the geographical localization of CTB within the 3D structure and confirm its directionality. The moderate expression of the EVT-specific marker HLA-G within our population implied that some portion of hiPSC were differentiating into EVT. The application of differentiation protocol variations, such as the two-step protocols developed recently, could be a way to further control STB and EVT lineage commitment and develop more specific functional models of early placental processes.^42^^,^^96^ Furthermore, future studies comparing our dataset to early trophoblasts will be needed to fully confirm the hypothesis that our model replicates early stages of placental development.

Although a fast, spontaneous syncytialization without the need for a treatment brings benefits in term of physiological relevance and can be viewed as an advantage, particular studies requiring better control over syncytialization may benefit more from inducible syncytialization models as is the case with cancerous trophoblast cell lines.

Finally, we focused on one human cell line to carefully characterize the 2D vs. microfluidic differentiation. Although the differentiation method described here showed consistent results across multiple independent experiments, future studies in hiPSC from different individuals would be needed to confirm the robustness of the differentiation and assess the effects of hiPSC from diseased patients on placental functions in the model.

## STAR★Methods

### Key resources table

**Table:** 

REAGENT or RESOURCE	SOURCE	IDENTIFIER
**Antibodies**		

Rabbit polyclonal anti-Nanog	Abcam	Cat#ab21624
Mouse monoclonal anti-Cytokeratin 7 (OV-TL 12/30)	ThermoFisher	Cat#MA5-11986; RRID: AB_10989596
Goat polyclonal anti-GATA-3	R&D Systems	Cat#AF2605
Mouse monoclonal anti-hCG beta (5H4-E2)	Abcam	Cat#ab9582
Rabbit monoclonal anti-E-Cadherin (24E10)	Cell Signaling Technology	Cat#3195
Donkey anti-Goat IgG (IRDye® 800CW)	LI-COR Biosciences	Cat#926–32214; RRID: AB_621846
Goat anti-Mouse IgG (IRDye® 680RD)	LI-COR Biosciences	Cat#926–68070; RRID: AB_10956588
Goat anti-Rabbit IgG (IRDye® 800CW)	LI-COR Biosciences	Cat#926–32211; RRID: AB_621843
Goat anti-Mouse IgG H&L (Alexa Fluor® 488)	Abcam	Cat#ab150113
Goat anti-Rabbit IgG H&L (Alexa Fluor® 594)	Abcam	Cat#ab150080
Donkey anti-Mouse IgG H&L (Alexa Fluor® 647)	ThermoFisher	Cat#A-31571
Goat anti-Rabbit IgG H&L (Alexa Fluor® Plus 488)	ThermoFisher	Cat#A32731

**Chemicals, peptides, and recombinant proteins**		

Geltrex™ LDEV-Free, hESC-Qualified, Reduced Growth Factor Basement Membrane Matrix	ThermoFisher	Cat#A1413301
Recombinant Human BMP-4 (E.coli derived)	PeproTech	Cat#120-05ET
PD 173074	Sigma-Aldrich	Cat#P2499
A 83-01	Tocris Bioscience	Cat#2939
NaHCO_3_	Sigma-Aldrich	Cat#S5761
Cultrex 3D Culture Matrix Rat Collagen I	R&D Systems	Cat#3447-020-01
Fluorescein isothiocyanate-Dextran, 10 kDa	Sigma-Aldrich	Cat#FD10S
Tetramethylrhodamine isothiocyanate-Dextran, 155 kDa	Sigma-Aldrich	Cat#T1287

**Critical commercial assays**		

RNeasy Mini Kit	Qiagen	Cat#74104
High-Capacity cDNA Reverse Transcription Kit	ThermoFisher	Cat#4368814
PowerUp SYBR Green Master Mix	ThermoFisher	Cat#A25742
Cell Lysis Buffer (10X)	Cell Signaling Technology	Cat#9803
DC™ Protein Assay Kit II	Bio-rad	Cat#5000112

**Deposited data**		

RNAseq data	This study	https://doi.org/10.5281/zenodo.7510757
Experimental Model and Study Participant Details		
ChiPS4 hiPSC cell line	Cellartis	N/A

**Oligonucleotides**		

QuantiTect GAPDH primers for qPCR	Qiagen	Cat#249900
Other primers for qPCR: see Table S1	See Table S1	N/A

**Software and algorithms**		

QuantStudio™ Software v1.3	https://www.thermofisher.com/fr/fr/home/global/forms/life-science/quantstudio-6-7-flex-software.html	N/A
GraphPad Prism 9	https://www.graphpad.com/scientific-software/prism/	N/A
Image Studio Lite™ acquisition software	https://www.licor.com/bio/image-studio-lite/	N/A
LAS X 3D software v4.2.0	https://www.leica-microsystems.com/products/microscope-software/p/leica-las-af-3d-visualization/	N/A
Fiji (ImageJ)	https://imagej.nih.gov/ij/download.html	N/A
Subread package v1.5.2	https://subread.sourceforge.net/	N/A
DESeq2	https://bioconductor.org/packages/release/bioc/html/DESeq2.html	N/A
Morpheus	https://software.broadinstitute.org/morpheus/	N/A
STRING	https://string-db.org/	N/A

**Other**		

OrganoPlate® 3-lane 40	Mimetas	Cat#4003-400B
OrganoFlow®	Mimetas	Cat#MI-OFPR-L
Incucyte S3 Live-Cell Analysis instrument	Sartorius	N/A

### Resource availability

#### Lead contact

Further information and requests for resources and reagents should be directed to and will be fulfilled by the lead contact, Colin E. Murdoch (c.z.murdoch@dundee.ac.uk)

#### Materials availability

This study did not generate new unique reagents. Information on reagents used in this study is available in the key resources table.

### Experimental model and study participants

#### hiPSC cell line

The human iPSC line ChiPS4 (Takara Bio) is derived from commercially available primary human dermal fibroblast cell lines (CCD-1112SK, CRL-2429, ATCC) by reprogramming using a polycistronic lentiviral vector expressing the Yamanaka factors (Klf-4, Oct-4, Sox-2, c-Myc). They were generated, routinely maintained and provided by Dr Lindsay Davidson (Human Pluripotent Stem Cell Facility, University of Dundee, UK). Undifferentiated ChiPS4 were maintained on Geltrex (ThermoFisher) coated plates (10 μg/cm^2^) using mTeSR medium containing 30 ng/mL FGF-2 and 10 ng/mL Noggin at 37°C with 5% CO_2_.^97^ Culture media was replenished daily and cells were passaged twice a week. Briefly, cells were exposed to TrypLE select (ThermoFisher) for 5 min at 37°C, resuspended in culture medium further supplemented with 10 μM Y-27632 (Tocris Bioscience) and seeded at a density of 3-5x10^4^ cells/cm^2^.

### Method details

#### Trophoblast differentiation in the microfluidic device

The protocol used for trophoblast differentiation was adapted from Amita et al.^41^ OrganoPlate 3-lane 40 (Mimetas) were used for ChiPS4 culture. An ECM scaffold was made by mixing 1M HEPES (ThermoFisher), 37 g/L NaHCO_3_ (Sigma-Aldrich) and 5 mg/mL rat collagen-I (R&D Systems) on ice at a 1:1:8 ratio. 2 μL were loaded per chip in the middle channel inlet and plates were incubated at 37°C and 5% CO_2_ for 15 min. A coating solution was made by diluting Geltrex in cold DMEM to a final concentration of 0.2 mg/mL 40 μL of coating solution were loaded the top channel inlets and plates were incubated at 37°C and 5% CO_2_ for 1 h. The wet channel seeding with passive pumping protocol was followed according to the manufacturer’s instructions. Before seeding cells, the coating solution was aspirated and top channels washed with PBS. ChiPS4 were resuspended at 10 000 cells/μL in mTeSR media containing 30 ng/mL FGF-2 and 10 μM Y-27632. 50 μL of medium was added to top channel outlets and 2 μL of cell suspension were loaded in top channel inlets. Plates were placed on a plate stand (Mimetas) for 4 h. 50 μL of medium were added to top channel inlets and plates were placed on an OrganoFlow interval rocker platform (Mimetas) set at a 7-degree inclination and 8-min cycle time. After 24 h, the medium was changed to mTeSR containing 20 ng/mL FGF-2. After a further 24 h, the medium was changed to mTeSR lacking FGF-2 and added with 10 ng/mL BMP4, 1 μM A83-01 (Tocris Bioscience) and 0.1 μM PD173074 (Sigma-Aldrich) (here referred to as BAP) and replenished daily until the experiment was terminated. Phase-contrast images were taken using the Incucyte S3 Live-Cell Analysis system (Sartorius). For control cultures differentiated in 2D conditions, ChiPS4 cells were seeded in 12-well plates coated with 10 μg/cm^2^ Geltrex at a density of 18 000 cells/cm^2^, exposed to similar culture and differentiation media as in the OrganoPlate platform and cultured under static conditions.

#### Quantitative reverse transcription-polymerase chain reaction (RT-qPCR)

Cells were lysed in buffer RLT (Qiagen) and lysates were homogenised through a Qiashredder spin column (Qiagen). Total RNA was extracted using RNeasy Mini Kit (Qiagen) in accordance with the manufacturer’s instructions. For each sample, RNA concentration and absorbance ratios were determined using a Nanodrop. Equal amounts of RNA were used as a template to synthesize cDNA using the high-capacity cDNA reverse transcription kit (ThermoFisher) in accordance with the manufacturer’s instructions. qPCR reactions were made with 1X PowerUp SYBR green Master Mix (ThermoFisher) and RT-qPCR analysis was performed on a QuantStudio 7 Flex real-time PCR system (Applied Biosystems) following standard cycling mode. Primers used for RT-qPCR amplification are described in Table S8.

#### RNA-seq

RNA was harvested from independent samples of ChiPS4 cultured in the OrganoPlate device or in conventional 2D culture plates before or after 4 days of BAP treatment using RNeasy Mini Kit (Qiagen) in accordance with the manufacturer’s instructions with in-column DNase digestion. Library preparation and sequencing were performed by Azenta Life Sciences. Library preparation was performed using Poly(A) selection and the Illumina NovaSeq, 2x150 platform was used for sequencing.

#### Western blot analysis

Whole-cell extracts were prepared with cell lysis buffer (Cell Signaling Technology) comprised of 20 mM Tris-HCl (pH 7.5), 150 mM NaCl, 1 mM Na_2_EDTA, 1 mM EGTA, 1% Triton X-100, 2.5 mM sodium pyrophosphate, 1 mM beta-glycerophosphate, 1 mM Na_3_VO_4_, 1 μg/mL leupeptin and freshly added with protease inhibitor cocktail (cOmplete). Cell lysates were sonicated using Vibra-Cell sonicator (Sonics and Materials) for 10 s at a 25% amplitude (5s on/5s off pulse) and centrifuged at 14,000 g for 10 min at 4°C. The supernatant was collected and used for western blotting. The proteins were quantified using the DC protein assay kit (Bio-Rad) in accordance with the manufacturer’s instructions. Proteins (20 μg) were mixed with 1X lithium dodecyl sulfate (LDS) sample buffer (ThermoFisher) and 5% β-mercaptoethanol, heated at 90°C for 10 min and separated by SDS-PAGE at 130V for 1 h using Bolt 8–12% Bis-Tris Plus gels (ThermoFisher) in MOPS SDS running buffer (ThermoFisher). Eluted proteins were transferred to 0.45 μm pore size Amersham nitrocellulose membranes (GE Healthcare Life Sciences) using a XCell II Blot Module (Novex) at 30V for 1 h. Blots were blocked with ROTIBlock (Carl Rot) and incubated overnight with the following primary antibodies: anti-Cytokeratin 7 (1:100, Cat#MA5-11986), anti-GATA-3 (1:800, Cat#AF2605) and anti-Nanog (1:200, Cat#ab21624). The next day, membranes were incubated for 1 h with the following secondary antibodies: donkey anti-goat IgG (IRDye 800CW, 1:20,000, Cat#926–32214), goat anti-mouse IgG (IRDye 680RD, 1:20,000, Cat#926–68070) or goat anti-rabbit IgG (IRDye 800CW, 1:20,000, Cat#926–32211). Fluorescence at 700CW and 800CW was detected using Odyssey CLx (LI-COR Biosciences). Total protein was visualised using Revert 700 Total Protein Stain (LI-COR Biosciences) according to the manufacturer’s instructions. Image Studio Lite was used for gel image acquisition.

#### Immunostaining

For OrganoPlate experiments, culture medium was aspirated from top channels, cells were fixed in 4% PFA for 15 min and rinsed twice in PBS. Chips were washed with 2% FBS in PBS and permeabilised in 0.3% Triton X-100 for 10 min. After another wash, cells were blocked in 2% FBS, 2% BSA, 0.1% Tween 20 in PBS for 45 min and incubated overnight at 4°C under static conditions with the following primary antibody solutions in blocking buffer: anti-Cytokeratin 7 (1:200, Cat#MA5-11986), anti-E-Cadherin (1:200, Cat#3195S) or anti-β-hCG (1:200, Cat#ab9582). The next day, chips were washed 3 times with 2% FBS in PBS and incubated with the following secondary antibody solutions made in blocking buffer + Hoechst 33342: donkey anti-mouse (Alexa Fluor 647, 1:250, Cat#A-31571) or goat anti-rabbit (Alexa Fluor Plus 488, 1:250, Cat#A32731) for 30 min at room temperature on the OrganoFlow set at a 7-degree inclination and 2-min cycle time. Plates were washed twice with 2% FBS in PBS and once with PBS before image acquisition. Images were acquired using a Molecular Devices ImageXpress Micro Confocal High-Content Imaging System (Molecular Devices, San Jose, CA, USA).

Confocal images were acquired on a Leica Stellaris 8 FALCON/FLIM system supplied with a Diode 405 nm laser and White Light Laser (WLL), tuneable from 440 to 790 nm. Images were acquired with an HC PL APO CS2 20x/0.75 lens using a pinhole size set to 56.6 μm (1 AU), a scan speed of 400 Hz and pixel dwell time of 3.16μs and averaging set to 5. Imaging was performed in two sequential line scans. First, DAPI was imaged with the 405-diode laser using a HyD S1 detector emitted light was collected with a gain of 103.4 collecting 425–718 emitted light. Secondly, Alexa Fluor 488 and Alexa Fluor 647 were excited with the WLL at 499 nm and 653 nm respectively. AF488 emitted light was collected on a HyD S detector with a spectral window of 504–620 nm. AF647 emitted light was collected on a HyD X detector with a spectral window of 659–750 nm. Z-stacks of approximately 180 μm total thickness were acquired with a step size of 0.686 μm. Gain and laser power were compensated to achieve a comparable dynamic range throughout the z stack. Z-stacks were converted into 3D reconstructions using LASX3D software version 4.2.0.

For immunostaining in 2D conditions, cells were cultured on 8-well μ-slides (Ibidi) and fixed in 4% PFA for 10 min followed by a post-fixation step in 90% methanol for 5 min. Fixed cells were blocked in 4% BSA, 0.2% Triton X-100 in PBS for 1 h and incubated overnight with the following primary antibody solutions in blocking buffer: anti-NANOG (1:100, Cat#ab21624) or anti-Cytokeratin 7 (1:100, Cat#MA5-11986). The next day, cells were incubated for 1 h with the following secondary antibody solutions made in blocking buffer: goat anti-mouse IgG (Alexa Fluor 488, 1:200, Cat#ab150113) or goat anti-rabbit (Alexa Fluor 594, 1:200, Cat#ab150080). Two drops of Vectashield mounting solution containing DAPI (Vector Laboratories) was added to each well and slides were imaged using a DeltaVision CoreDV Widefield deconvolution microscope.

#### Barrier integrity assay

Fluorescent working solutions were made by reconstituting Fluorescein isothiocyanate (FITC)-dextran, 10 kDa (Sigma-Aldrich) or Tetramethylrhodamine isothiocyanate (TRITC)-dextran, 155k Da (Sigma-Aldrich) at 25 mg/mL in Hank’s balanced salt solution. A wetting step was performed before each assay by adding 50 μL mTeSR medium to all inlets and outlets and placing the plate on a 7-degree inclination for 5 min. The medium was aspirated from all inlets and outlets and 20 μL was added in the middle and bottom inlets and outlets. 40 μL and 30 μL of fluorescent working solutions diluted at 1:50 in mTeSR medium were added to the top channel inlet and outlet respectively and image acquisition was started within 2 min. Plates were imaged every 2 min for 10 min using the Incucyte S3 Live-Cell Analysis instrument (Sartorius) with exposure times of 200 ms and 600 ms for the green and the red channel respectively. Mean fluorescence signals in the top and middle channels were quantified on ImageJ and plotted on GraphPad Prism to calculate areas under the curve values. For each independent experiment, two different chips were taken into account in the ratio calculation as previously described.^98^

### Quantification and statistical analysis

#### Analysis of RT-qPCR data

RT-qPCR data were acquired using the QuantStudio Software v1.3 and gene expression was quantified using the standard 2^−ΔΔCt^ method. The expression levels of target genes were normalised to that of *GAPDH* throughout the study.

#### Analysis of RNA-seq data

Trimming, mapping and differential gene expression analysis were performed by Azenta Life Sciences. Sequence reads were trimmed to remove possible adapter sequences and nucleotides with poor quality using Trimmomatic v.0.36. The trimmed reads were mapped to the Homo sapiens GRCh38 reference genome available on ENSEMBL using the STAR aligner v.2.5.2b. Unique gene hit counts were calculated by using featureCounts from the Subread package v.1.5.2. and used for downstream differential expression analysis using DESeq2. The Wald test was used to generate p values and log2 fold changes, and a Benjamini-Hochberg test to calculate adjusted p values. Genes with an adjusted p value <0.05 and absolute log2FoldChange >1 were called differentially expressed genes for each comparison. Normalised read counts from literature curated custom gene sets of hiPSC, trophectoderm, ectoderm, mesoderm, endoderm, CTB, EVT and STB markers were loaded onto Morpheus to generate heatmaps.^39^^,^^47^^,^^48^^,^^49^^,^^50^^,^^51^^,^^52^^,^^56^^,^^57^^,^^58^ The gene ontology (GO) and Reactome pathway analyses were performed on the STRING platform and gene lists from the DEseq2 tables were loaded as reference genes.^99^

## Data Availability

All datasets are accessible in public databases• RNAseq dataset (fastq files) have been deposited with Zenodo community iPlacenta https://zenodo.org/search?page=1&size=20&q=iplacenta under accession number https://doi.org/10.5281/zenodo.7510757 • Data has been deposited at https://doi.org/10.17632/wrxsr5drvy.1 • Original microscopy data can be accessed at OMERO on request.

## Supplemental information

Document S1. Figures S1–S3, Table S8, and Data S1–S4

Table S1. Comparative expression analysis of lineage markers in BAP-derived trophoblasts before and after 4 days of BAP treatment in the microfluidic platform, related to Figure 2 Normalized read counts were calculated from RNA-seq data on RNA samples from independent experiments. Genes that differed significantly in expression (log2FoldChange > |1| and padj <0.05) between day 0 and day 4 are marked with ∗. Genes referred to as “not detected” showed an average read count <10 across all samples tested and were therefore filtered out before normalization and downstream differential expression analysis.

Table S2. Comparative expression analysis of cytotrophoblast, syncytiotrophoblast and extravillous trophoblast signature genes in BAP-derived trophoblasts before and after 4 days of BAP treatment in the microfluidic platform, related to Figure 3 Normalized read counts were calculated from RNA-seq data on RNA samples from independent experiments before and after 4 days of BAP treatment. Genes referred to as “not detected” showed an average read count <10 across all samples tested and were therefore filtered out before normalization and downstream differential expression analysis. Genes that differed significantly in expression (log2FoldChange > |1| and padj <0.05) between day 0 and day 4 are marked with∗.

Table S3. List of selected genes associated with major STB functions significantly upregulated in the 4-day hiPSC-derived placenta-on-chip, related to Figure 3 Normalized read counts were calculated from RNA-seq data on RNA samples from independent experiments before and after 4 days of BAP treatment.

Table S4. List of ABC and SLC transporters significantly upregulated in the 4-day hiPSC-derived placenta-on-chip, related to Figure 4 Normalized read counts were calculated from RNA-seq data on RNA samples from independent experiments before and after 4 days of BAP treatment.

Table S5. List of differentially expressed genes in hiPSC-derived trophoblasts cultured in the microfluidic platform compared to cells differentiated in 2D conditions, related to Figure 5 Normalized read counts were calculated from RNA-seq data on RNA samples from independent experiments after 4 days of BAP treatment. Genes showing log2FoldChange > |1| and padj <0.05 were considered as differentially expressed.

Table S6. List of GO terms and Reactome pathways enriched in DE genes between microfluidic and 2D differentiation, related to Figure 5 Over-representation analysis was performed by uploading the list of total DE genes (1,019), significantly upregulated (462) or down-regulated (557) genes expressed by hiPSC-derived trophoblasts differentiated in the microfluidic device compared to the conventional 2D culture plate on the STRING platform.

Table S7. Expression changes of DE genes involved in ECM organization, Enzyme-linked receptor protein signaling or type I interferon signaling between hiPSC-derived trophoblasts differentiated in a microfluidic device compared to the conventional culture plate, related to Figure 5 Normalized read counts were calculated from RNA-seq data on RNA samples from independent experiments before and after 4 days of BAP treatment. Main figure titles and legends.

Figure 360. An author presentation of Graphical abstract
