# Supplementary material for: Development of a human iPSC-derived placental barrier-on-chip model
Source: iScience. 2023 Jul 13;26(7):107240. doi: 10.1016/j.isci.2023.107240 (PMC10392097; doi:10.1016/j.isci.2023.107240)

iScience, Volume *26*

Supplemental information

Development of a human iPSC-derived placental barrier-on-chip model

Agathe Lermant, Gwenaëlle Rabussier, Henriëtte L. Lanz, Lindsay Davidson, Iain M. Porter, and Colin E. Murdoch

Figure S1: iPSC-derived trophoblasts form a leak-tight barrier when differentiated in a microfluidic device, related to Figure 4.

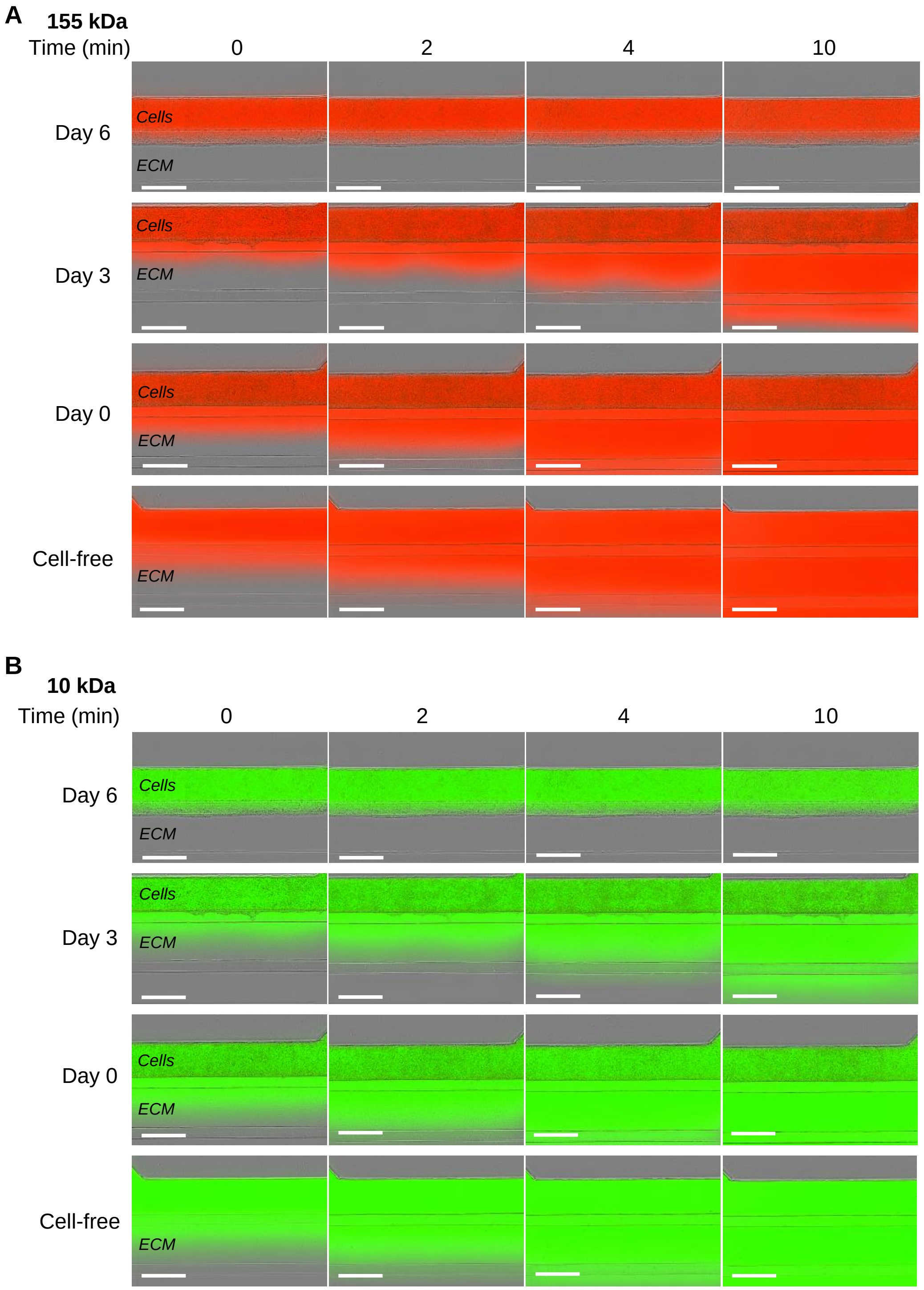

Representative images of fluorescent signal 2, 4 and 10 minutes after adding 155 kDa TRITC-Dextran (A) or 10 kDa FITC-Dextran (B) compounds in the top channel of day 0, 3 and 6 cultures compared to a cell-free channel. Scale bar, 400 µm.

Figure S2: STRING network showing protein-protein associations in the list of genes down-regulated upon microfluidic differentiation, related to Figure 5.

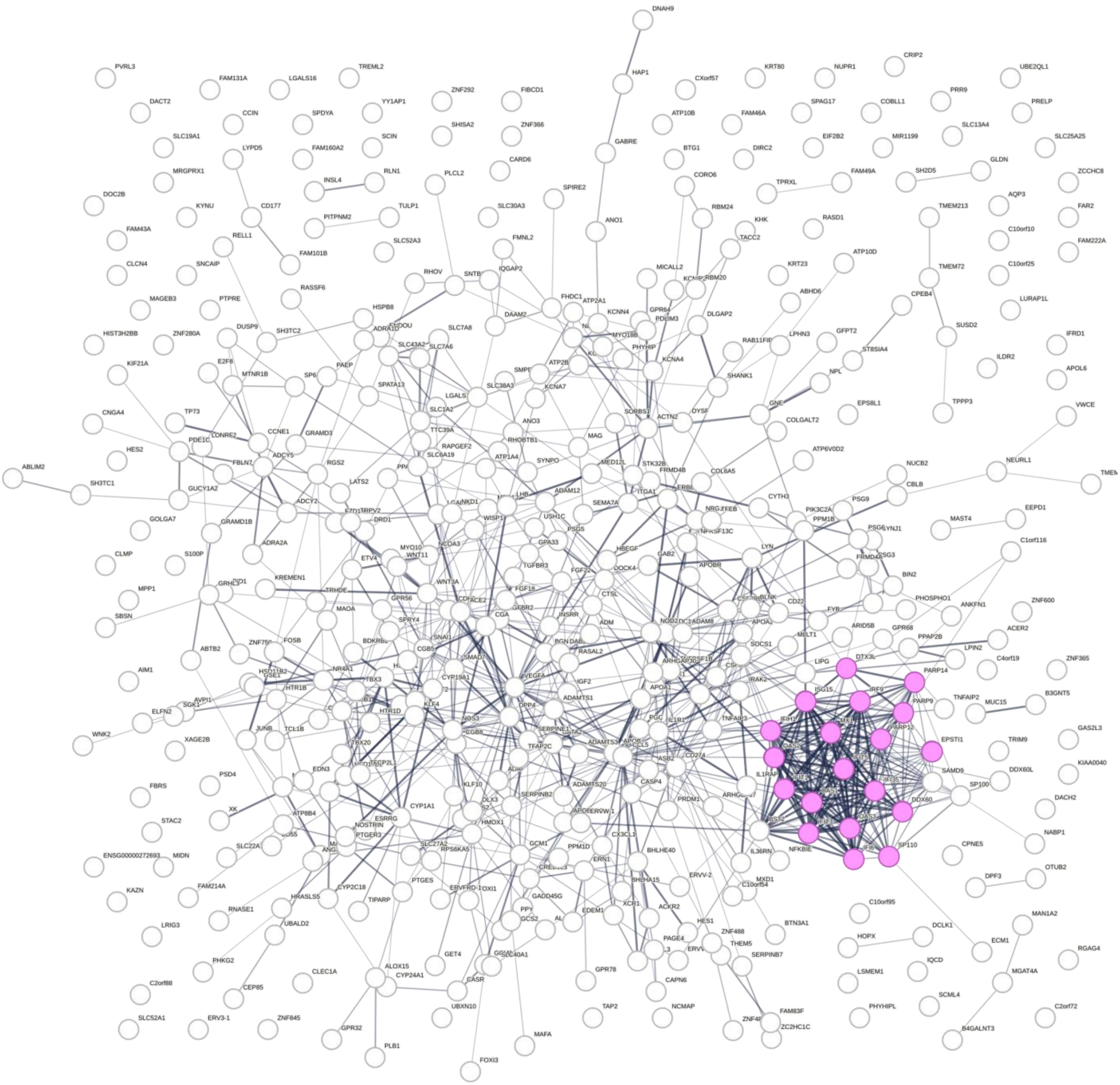

Nodes represent proteins, and edges represent protein-protein associations. For each association, edge confidence is proportional to the edge thickness. Coloured nodes represent a local cluster of proteins associated with Interferon alpha/beta signalling and negative regulators of DDX58/IFIH1 signalling.

Figure S3: Heatmaps showing relative expression of CTB, STB and EVT-associated genes before (Day 0) and after 4 days of BAP treatment, sorted by padj and added with corresponding gene ID, related to Figure 3.

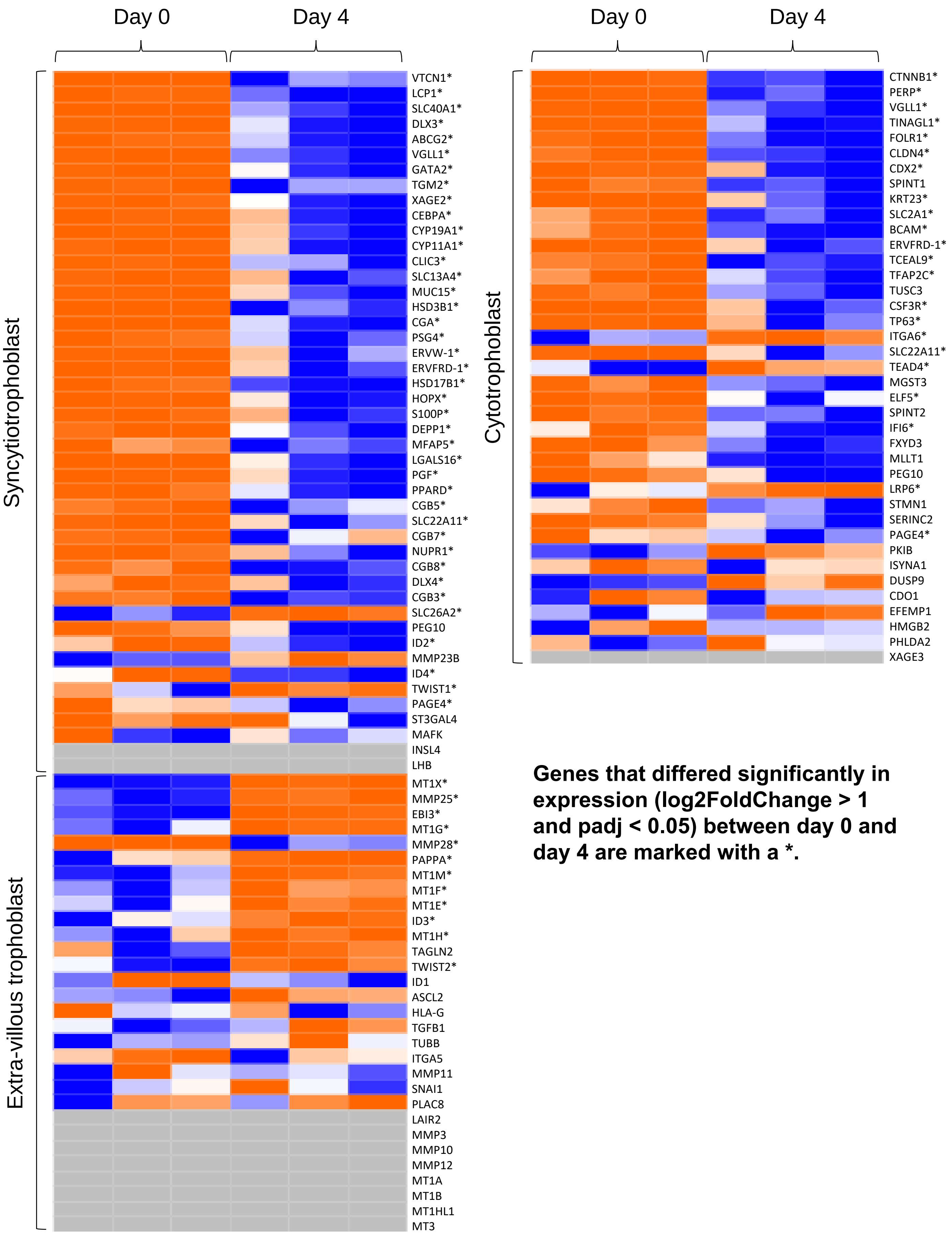

Genes that differed significantly in expression (log2FoldChange > 1 and padj < 0.05) between day 0 and day 4 are marked with a *.

Table S8: List of primers used for RT-qPCR, related to Figure 1, Figure 2 and Figure 3.

Target gene	Forward primer (5' -> 3')	Reverse primer (5' -> 3')
KRT-7	AAGAACCAGCGTGCCAAGT	TCCAGCTCCTCCTGCTTG
GATA3	TGCAGGAGCAGTATCATGAAGCCT	GCATCAAACAACTGTGGCCAGTGA
PGF	CCTACGTGGAGCTGACGTTCT	TCCTTTCCGGCTTCATCTTCT
HLA-G	ACAACCAGAGCGAGGCCAGT	AGACAGGGTGGTGGGTCACG
CGB	AGCACTTTGCTCGGGTCACGG	TGGTCCAGCGCCAAGGGTGA
ERVW-1	CTACCCCAACTGCGGTTAAA	GGTTCCTTTGGCAGTATCCA
ERVFRD-1	CCAAATTCCCTCCTCTCCTC	CGGGTGTTAGTTTGCTTGGT
HLA-A	CAGACGCCGAGGATGGCC	CACACAAGGCAGCTGTCTCACA
NANOG	CATGAGTGTGGATCCAGCTTG	CCTGAATAAGCAGATCCATGG
POU5F1	GACAACAATGAGAACCTTCAGGAGA	CTGGCGCCGGTTACAGAACCA
TBXT	CCTTGCTCACACCTGCAGTAGC	GGCCAACTGCATCATCTCCA

Data S1 : Full immunoblot for GATA3 related to figure 1

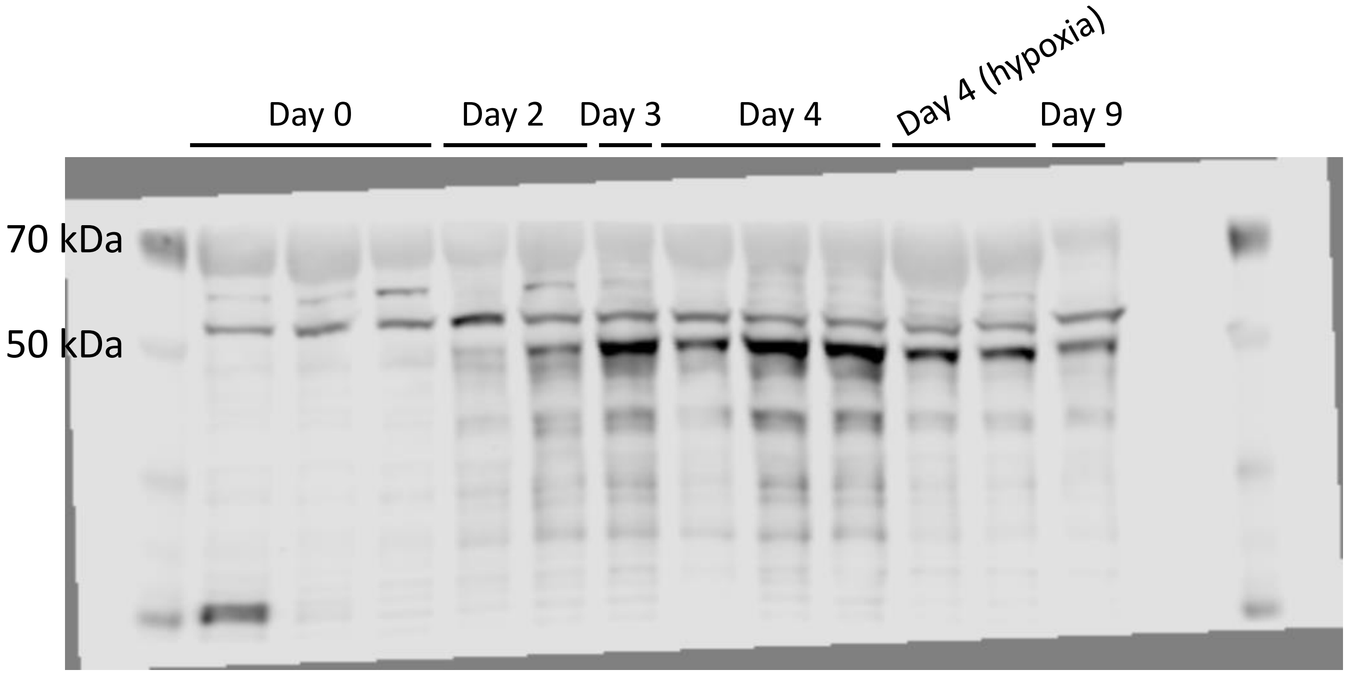

Data S2: Full immunoblot for NANOG related to figure 1

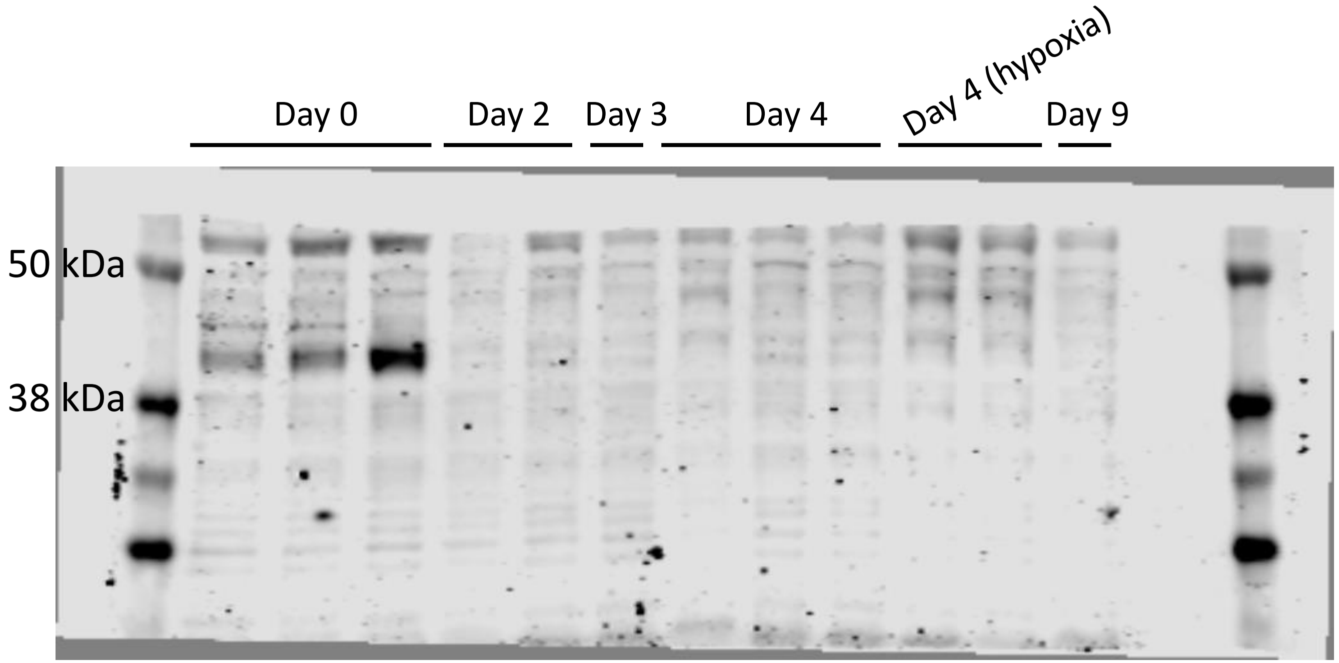

Data S3 : Full immunoblot for KRT-7 related to figure 1

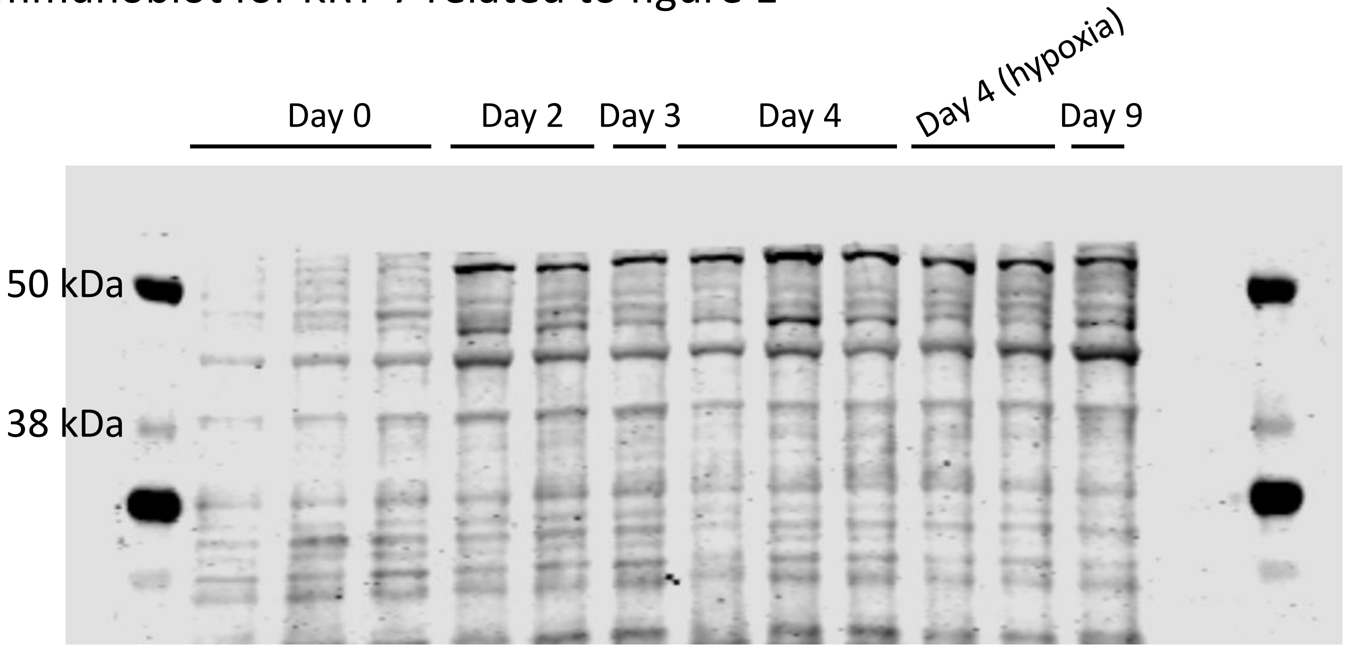

Data S4 : Full immunoblot for KRT-7 related to figure 1

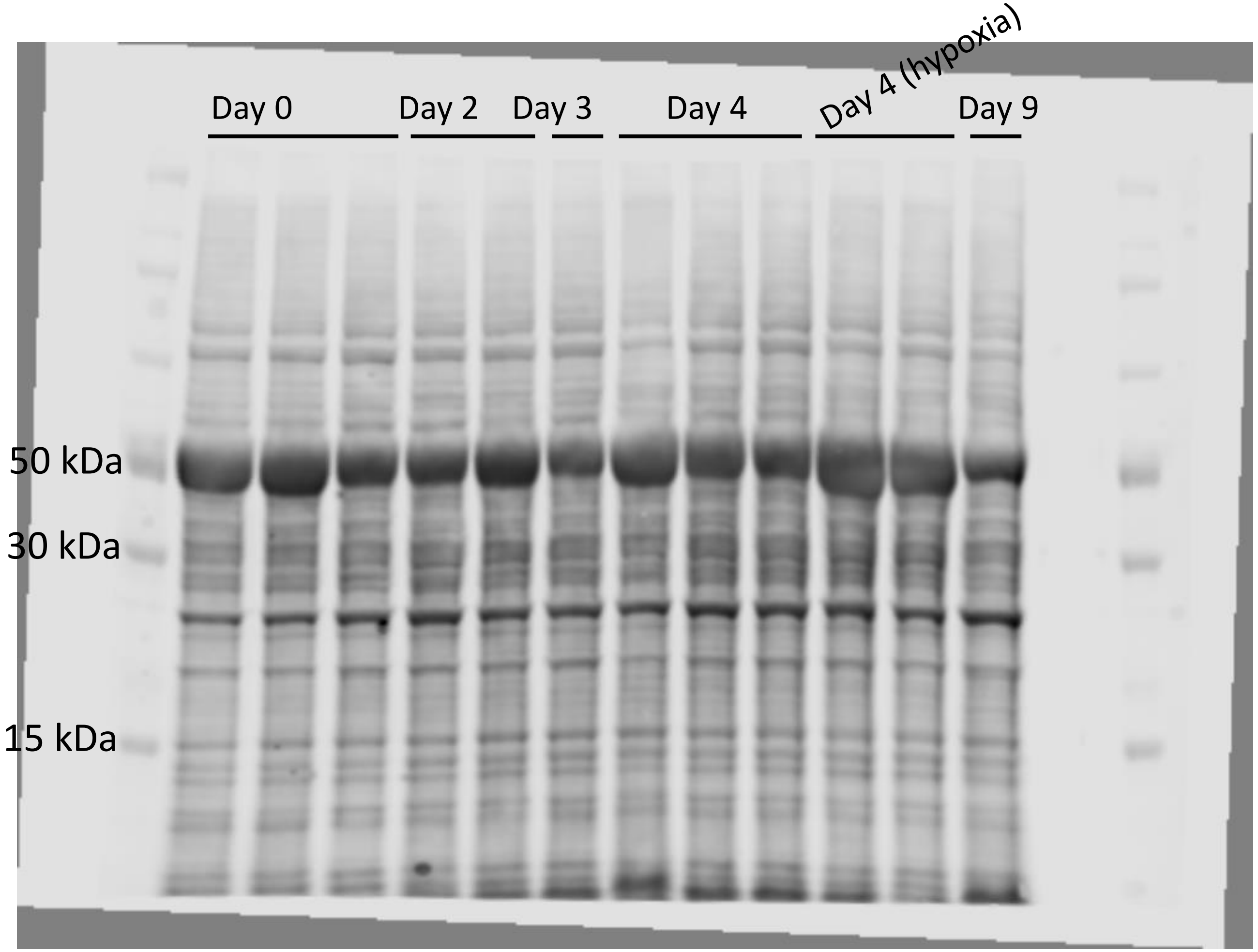

Supplement: Document S1. Figures S1–S3, Table S8, and Data S1–S4 [file mmc1.pdf]
